# Identifying genetic markers associated with susceptibility to cardiovascular diseases

**DOI:** 10.4155/fsoa-2018-0031

**Published:** 2018-10-26

**Authors:** Hitesh Shukla, Jessica Louise Mason, Abdullah Sabyah

**Affiliations:** 1 Rightangled Ltd, The Relay Building, 114 Whitechapel High St, London E1 7PT, UK

**Keywords:** cardiovascular disease, DNA, genetic, genotyping, heart, medicine, personalised, pharmacogenetics, polymorphism, SNP

## Abstract

The development of cardiovascular diseases (CVDs) is due to a complex interaction between the genome and the environment. Understanding how genetic differences in individuals contribute to their susceptibility to CVDs can help guide practitioners to give the best advice to achieve a favorable outcome for the patient. As genome technologies evolve, genotyping of individuals could be available to all patients using a simple saliva test. Large-scale genome-wide association studies and meta analyses have provided powerful insights into polymorphisms that may be predictive of disease and an individual's response to certain nutrients, but moving forward it is imperative that these insights can be applied in the medical setting to reduce the incidence and mortality of CVDs.

## Lay abstract

Cardiovascular diseases (CVDs) are the leading cause of death worldwide, and while most CVDs can be prevented by adopting a healthy lifestyle, this is only half the story. Evidence suggests changes in an individual's genes or DNA can cause some form of CVDs, highlighting a complex relationship between genes and the environment. Genotyping, a process used to determine genetic differences within an individual's DNA, can provide doctors with relevant information to identify individuals who are at high risk of developing CVDs. This would allow treatment to begin early and encourage individuals to adopt a healthy lifestyle to reduce their risk.

Cardiovascular diseases (CVDs), characterized by diseases of the heart and blood vessels, are the number one cause of death globally, representing 31% of all deaths in 2015 [1]. Most CVDs can be prevented by a healthy lifestyle, including a healthy diet, regular physical activity, maintenance of a healthy weight, not smoking and not drinking excessively. These factors will influence the intermediate risk factors of CVD, which include blood pressure, blood lipid concentration, blood glucose levels and obesity. However, as with most diseases, the development of many CVDs is a complex interplay of the effect of an individual's genome and their environment. There is strong evidence that proves there is indeed a genetic cause of CVDs, such as coronary artery disease (CAD), which has an estimated heritability of 40–50% [2]. On the other hand, some diseases such as sickle cell anemia (SCA) are known to be caused by genetics only, with environmental factors having no effect [3]. However, most diseases arise due to the complicated interaction between genes and the environment, and many of these interactions have not been fully elucidated.

Consequently, over the past 10 years, many studies have been conducted to identify genes and genetic variants involved in the pathogenesis of CVDs, and the precise mechanism by which these exert their effect on an individual. Since the Wellcome Trust Case Control Consortium genome-wide association study (GWAS) in 2007, and the hundreds of GWAS studies conducted since, many single nucleotide polymorphisms (SNPs) have been associated with increased risk of developing CVDs and with influencing conditions that can contribute to CVD risk including blood lipid levels, obesity and hypertension [4–6].

Currently, an individual's risk of developing CVD can be assessed by their lifestyle factors, such as physical activity levels, diet and smoking; but this does not consider the genetic susceptibility to CVDs. When an individual is classed as an intermediate risk based on these lifestyle factors, it can be hard to determine if they will progress into developing CVDs, which is where genotyping could become extremely useful [7]. Importantly, genetic data could also be used to determine if individuals would benefit from early interventions to prevent the development of CVDs [8]. In a recent study conducted by Khera *et al*., individuals with a high genetic risk for CAD had a 91% higher risk of cardiac events compared with those with a lower risk genetic profile [9]. This study also demonstrated that, despite this genetic risk, a healthy lifestyle contributed to a 46% reduction in the risk of cardiac events, compared with those with a less favorable lifestyle, showing that genotyping for risk of CVDs could have a real impact on the reduction of cardiac events [9]. This suggests that an individual's genotype could be assessed relatively inexpensively when they are asymptomatic to prevent CVD, which has a significant cost to health services and individuals once it progresses and has fatal outcomes [10]. Therefore, it is imperative that genetic testing is introduced within a clinical setting to not only reduce incidence and mortality of CVDs, but to promote prevention over treatment.

GWAS have also been useful in the development of potential new therapeutics to treat CVDs. The identification of a loss of function SNP in the gene encoding Proprotein Convertase Subtilisin/Kexin Type 9 (*PCSK9*) that reduced the risk for CAD and myocardial infarction by reducing LDL levels [11], led to the development of a monoclonal antibody therapy that targets the functional protein to treat hypercholesterolemia, which has shown success in statin-intolerant patients [12].

The identification of polymorphisms that confer an increased risk for CVDs, and those determining responses to CVD drugs, will become increasingly important as available -omics technologies take us forward into the personalized medicine era. As CVDs are responsible for the most mortality across the globe compared with any other disease, it is important to identify those at a high risk early, so specific drugs are prescribed, and lifestyle is altered.

The ‘Heart DNA Test’ service at Rightangled Ltd involves genotyping for saliva-derived DNA at a remote laboratory, from a commercially available saliva kit. This literature review will focus on the genetic markers used by the Heart DNA Test, to highlight their significance and ultimately how genetics can influence an individual's response to metabolize nutrients in relation to: high-density lipoprotein (HDL) cholesterol, low-density lipoprotein (LDL) cholesterol, triglyceride levels, folate metabolism and Gene X carrier status. Additionally, this literature review will also cover the importance of genetics in determining an individual's susceptibility to the following cardiovascular conditions: type III hyperlipoproteinemia (HLP), atrial fibrillation (AF), CAD, myocardial infarction, peripheral arterial disease (PAD), venous thrombosis (VT), SCA and risk factors such as hypertension.

## Methods

### Information sources & search

**Figure 1.**
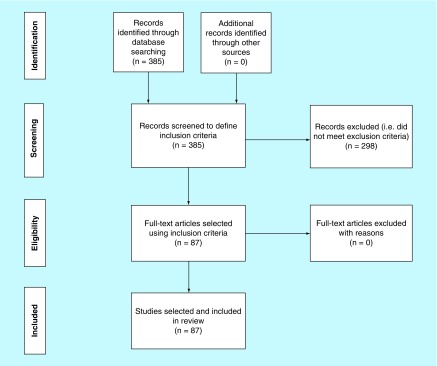
**Flowchart for study selection.** A systematic search of the PubMed, dbSNP and GWAS catalogs was carried out during the acquisition of data and preparation of manuscript phases. Search terms included gene name, accession number (rs number), parameter or condition (e.g., ‘high-density lipoprotein’, ‘low-density lipoprotein’, ‘Triglycerides’, ‘Coronary artery disease’, ‘Myocardial infarction‘) and/or ‘single nucleotide polymorphism’ and/or ‘Risk’ and/or ‘susceptibility.’ Search results were reviewed, and papers selected based on the following inclusion criteria: papers should mention gene or single nucleotide polymorphism of interest, single nucleotide polymorphisms that are significantly associated to the parameter or condition of interest and risk of cardiovascular disease.

Investigation of genetic markers was conducted by analyzing and reviewing existing markers in study articles across numerous databases including GWAS, meta-analysis studies, all databases in NCBI, MeSH in Medline and dbSNP. Databases such as EMBASE were not used in the search strategy due to an access fee being required. All relevant articles were identified by using search terms related to each of the conditions or genetic markers (SNPs) of interest (Figure 1).

### Study selection

Initial search involved the input of gene names and/or the markers of interest across the above databases. The resulting articles from the search criteria were title scanned to eliminate those having a distinct dissociation with the condition or marker of interest. Searches were conducted without the use of language restriction. Search entries in GWAS catalog resulted in articles that were selected based on their association count, reported trait, reported gene and SNP number, with additional associated SNPs being shortlisted for later review and potential inclusion in the final report depending on eligibility criteria. The reference list on the selected studies were screened to obtain additional sources of studies for review.

## Diet & CVD risk

Genetic markers can predispose an individual to metabolize certain nutrients differently to others, having an impact on CVD risk. The markers reviewed represent how genetics will influence cholesterol and triglyceride levels, folate metabolism and determining Gene X carrier status (Table 1). These markers are mostly analyzed in the context of influencing the development of atheromas, an event contributing to CAD and other conditions. Briefly, this process involves cholesterol packaging into HDLs and LDLs, known colloquially as ‘good’ and ‘bad’ cholesterol respectively. HDL removes excess cholesterol from cells, including arterial linings, where it is transported to the liver for further processing. LDL on the other hand delivers cholesterol to cells and can lead to lipid accumulation within the linings of blood vessels. This occurs when LDL crosses over the endothelial barrier and then becomes oxidized, before being taken up by macrophages. This process can be accelerated by endothelial damage, which can be caused by homocysteine, a toxic metabolite of irregular folate metabolism. Macrophages transform into foam cells (lipid loaded macrophages) by differentiation and emit growth factors leading to the migration of smooth muscle cells to the area. This structure is known as an atheroma and consists of lipids, foam cells and necrotic debris, which narrows the artery, termed atherosclerosis. As outlined in the sections below, an individual's unique blood lipid levels and homocysteine levels can be influenced by genetics as certain polymorphisms can lead to increased risk of CAD and other CVDs.

**Table 1.** **List of genetic markers that influence cholesterol levels, triglycerides levels, folate metabolism and Gene X carrier status**

**Parameter**	**Gene name**	**Genetic markers (SNPs)**	**Allele**	**Association**	**Ref.**
HDL	*ATP-binding cassette transporter 1 (ABCA1)*	rs1883025	A	Decreased HDL cholesterol levels	Kathiresan *et al*. (2009) [5] Cohen *et al*. (2004) [14] Willer *et al*. (2008) [15] Gupta *et al*. (2010) [16] Teslovich *et al*. (2010) [17] Papp *et al*. (2012) [18] Voight *et al*. (2012) [19] Oram (2003) [20]

	*Angiopoietin Like 4 (ANGPTL4)*	rs2967605	A	Decreased HDL cholesterol levels	

	*Fatty Acid Desaturase (FADS1)*	rs174547	C	Decreased HDL cholesterol levels	

	*Polypeptide N-Acetylgalactosaminyltransferase 2 (GALNT2)*	rs4846914	G	Decreased HDL cholesterol levels	

	*Hepatocyte nuclear factor 4 alpha (HNF4A)*	rs1800961	T	Decreased HDL cholesterol levels	

	*Potassium Channel Tetramerization Domain-Containing Protein 10 (KCTD10)*	rs2338104	C	Decreased HDL cholesterol levels	

	*Lecithin-Cholesterol Acyltransferase (LCAT)*	rs2271293	G	Decreased HDL cholesterol levels	

	*Hepatic triacylglycerol lipase (LIPC)*	rs10468017	T	Increased HDL cholesterol levels	

	*Endothelial Lipase (LIPG)*	rs4939883	T	Decreased HDL cholesterol levels	

	*Lipoprotein Lipase (LPL)*	rs12678919	A	Decreased HDL cholesterol levels	

	*Phospholipid transfer protein (PLTP)*	rs7679	C	Decreased HDL cholesterol levels	

	*Tetratricopeptide repeat domain 39B (TTC39B)*	rs471364	G	Decreased HDL cholesterol levels	

	*Zinc finger protein 259 (ZNF259)*	rs964184	G	Decreased HDL cholesterol levels	

LDL	*Low-density lipoprotein receptor (LDLR)*	rs6511720	T	Decreased LDL cholesterol levels	Kathiresan *et al*. (2009) [5] CARDIoGRAMplusC4D Consortium *et al*. (2013) [6] Yang *et al*. (2015) [22] Dumitrescu *et al*. (2011) [23]. Ganna *et al*. (2013) [24] Fairoozy *et al*. (2016) [25]

	*Transcription factor MafB (MAFB)*	rs6102059	T	Decreased LDL cholesterol levels	

	*Neurocan (NCAN)*	rs10401969	T	Increased LDL cholesterol levels	

	*Proprotein Convertase Subtilisin/Kexin Type 9 (PCSK9)*	rs11206510	T	Increased LDL cholesterol levels	

	*ATP Binding Cassette Subfamily G Member 8 (ABCG8)*	rs6544713	T	Increased LDL cholesterol levels	

	*Cadherin EGF LAG Seven-Pass G-Type Receptor 2 (CELSR2)*	rs12740374	T	Decreased LDL cholesterol levels	

	*3-Hydroxy-3-Methylglutaryl-CoA Reductase (HMGCR)*	rs3846663	T	Increased LDL cholesterol levels	

	*Hepatocyte nuclear factor 1-alpha (HNF1A)*	rs2650000	T	Increased LDL cholesterol levels	

	Intergenic	rs1501908	G	Decreased LDL cholesterol levels	

Triglycerides	*Angiopoietin Like 3 (ANGPTL3)*	rs10889353	C	Decreased Triglyceride levels	Kathiresan *et al*. (2009) [ 5] Scherag *et al*. (2010) [31] Varbo *et al*. (2010) [32] Santoro *et al*. (2011) [33] Zhang *et al*. (2012) [34] Mirhafez *et al*. (2016) [35] Kooner (2008) [36]

	*Apolipoprotein B (APOB)*	rs7557067	G	Decreased Triglyceride levels	

	*Fatty Acid Desaturase 1 (FADS1)*	rs174547	C	Increased Triglyceride levels	

	*Glucokinase regulatory protein (GCKR)*	rs1260326	T	Increased Triglyceride levels	

	*Lipoprotein Lipase (LPL)*	rs12678919	G	Decreased Triglyceride levels	

	*MLX Interacting Protein Like (MLXIPL)*	rs714052	C	Decreased Triglyceride levels	

	*Neurocan (NCAN)*	rs17216525	T	Decreased Triglyceride levels	

	*Phospholipid Transfer Protein (PLTP)*	rs7679	C	Increased Triglyceride levels	

	*Tribbles Pseudokinase 1 (TRIB1)*	rs2954029	T	Decreased Triglyceride levels	

	*XK-related protein 6 (XKR6)*	rs7819412	G	Decreased Triglyceride levels	

	*Zinc finger protein 259 (ZNF259)*	rs964184	G	Increased Triglyceride levels	

Folate	*Methylenetetrahydrofolate reductase (MTHFR)*	rs1801133	T	Decreased circulatory folate levels	Greenberg *et al*. (2011) [37] Czeizel *et al*. (2013) [38] Oakley *et al*. (1996) [39] Gao *et al*. (2012) [40] Frosst *et al*. (1995) [41]

Gene X	*Asialoglycoprotein Receptor 1 (ASGR1)*	del12	Carrier status	Carrier trait is associated with a 34% decrease in CAD risk.	Roggenbuck *et al*. (2012) [42] Li *et al*. (2008) [43] Nioi *et al*. (2016) [44]

ATP: Adenosine triphosphate; HDL: High density lipoprotein; LDL: Low density lipoprotein; SNP: Single nucleotide polymorphism.

### HDL cholesterol

A low blood HDL cholesterol level is the most common lipid abnormality in patients with premature development of atherosclerosis [13].

In various genome-wide studies and meta analyses, polymorphisms in the following genes have been shown to increase the risk of lower HDL cholesterol levels: *ABCA1*, *ANGPTL4*, *FADS1*, *GALNT2*, *HNF4A*, *KCTD10*, *LCAT*, *LIPC*, *LIPG*, *LPL*, *PLTP*, *TTC39B*, *ZNF259* [5,14–19]. These genes control various cellular pathways, including those involved in lipid metabolism and transport, so when their function varies due to a polymorphism, HDL cholesterol levels can be altered. The *ABCA1* gene, in particular, codes for the cellular adenosine triphosphate (ATP)-binding cassette transporter ABCA1 which has a key role in reverse cholesterol transport, where cellular cholesterol and phospholipids are removed from cells and packaged into lipoproteins that mature to form HDL particles. Polymorphisms in *ABCA1* result in impaired clearance of sterols from tissue, leading to accumulation in tissue macrophages, intestinal cells and hepatocytes [20].

To increase HDL levels, an individual's lifestyle should be altered. Some drugs are available to increase HDL, including niacin, which has the ability to increase HDL levels by up to 30% [21].

### LDL cholesterol

CAD risk is correlated with LDL levels, the higher the LDL cholesterol level, the higher the risk of CAD. LDL cholesterol levels can be high due to lifestyle factors, by underlying conditions such as hypertension and diabetes, and by genetics.

In various genome-wide studies and meta analyses, polymorphisms in the following genes have been shown to increase the risk of higher LDL cholesterol levels: *LDLR*, *MAFB*, *NCAN*, *ABCG8*, *PCSK9*, *CELSR2*, *HMGCR*, *HNF1A* [5,6,22–24]. These genes control lipid metabolism pathways, lipid packaging and lipid transport through the bloodstream, conferring an increased genetic risk of altered LDL cholesterol levels. An example of a known mechanism of action is the *LDLR* gene, which codes for the low-density lipoprotein receptor (LDLR) which has a key involvement in receptor-mediated endocytosis of LDL-C particles, removing them from the blood for further processing by the liver. A polymorphism in this gene causes an increase in LDLR expression, increasing LDL removal from the bloodstream, therefore reducing the risk of CAD [25].

An individual with a high-risk genotype should try to reduce LDL cholesterol levels through lifestyle changes. Drugs such as statins may be prescribed if lifestyle changes are ineffective or inapplicable, which lower LDL levels and consequently lower the risk of CAD, myocardial infarction, stroke, PAD and total mortality [26–28].

### Triglycerides

Triglycerides are a type of lipid found in the blood and are an essential energy supply to cells of the body, and can be stored as body fat if they are not immediately used. Elevated total triglyceride levels are a risk factor for CAD independent of HDL levels [29,30], and very high levels of triglycerides can cause acute pancreatitis.

Polymorphisms in certain genes have been linked to increased triglyceride levels in various genome wide studies and meta analyses. These genes include: *ANGPTL3*, *APOB*, *FADS1*, *GCKR*, *LPL*, *MLXIPL*, *NCAN*, *PTLP*, *TRIB1*, *XKR6*, *ZNF259* [5,31–35]. These genes are involved in lipid metabolism and transport. As an example, *MLXIPL* encodes a transcription factor with a role in glucose utilization and storage, controlling genes involved in glycolysis, lipogenesis, triglyceride synthesis and very LDL secretion in a glucose-dependent manner. Polymorphisms in this gene change the rate of triglyceride synthesis, with the wild-type allele conferring more efficient food utilization, fat deposition and weight gain, increasing risk of CAD [36]. To reduce triglyceride levels despite genetic risk, individuals should change their diet and adopt lifestyle changes. If triglyceride levels remain unchanged, medication may be recommended which could include statins, niacin or gemfibrozil.

### Folate

Folate is a natural B vitamin required for DNA and RNA construction, amino acid synthesis, vitamin metabolism and its role as a substrate for numerous enzymatic reactions [37]. Folate cannot be produced in the body and is therefore required through numerous food sources including leafy vegetables, juices, wheat bread and beans [38]. The *MTHFR* gene encodes the methylenetetrahydrofolate reductase (MTHFR) enzyme involved in processing amino acids and vitamin B9; 5-MTHFR specifically is the final substrate with crucial importance in mediating chemical reactions required for the conversion of the toxic metabolite homocysteine into methionine [39].

A single polymorphism within the *MTHFR* gene impacts the conversion of 5,10-methylene-THF to 5-MTHFR, the form required to efficiently process folate [38]. Homozygous forms (T/T) are associated with a 70% reduction in MTHFR activity thus contributing to decreased circulatory folate levels and impeded conversion of homocysteine to methionine. Increased levels of this toxic metabolite contribute to increased risk of stroke, blood vessel damage, VT and neural tube defects [38,40,41]. Treatments may involve vitamin B12 or B9 injections if the cause is found to be nondietary related.

### Gene X

The *ASGPR* gene codes for asialoglycoprotein receptors (ASGPR), a C-type lectin located on the surface of hepatic cells with a key responsibility in binding glycoproteins as well as its involvement in immunoglobulin clearance, clearance of LDL and the removal of apoptotic cells [42,43]. Calcium levels, pH levels and galactose residue positioning determine the success rate in which receptor-glycoprotein interaction occurs [42]. Once a receptor–ligand complex is established, receptor-mediated endocytosis is initiated, followed by the lysosomal degradation of glycoproteins [44].

A recent study by Nioi *et al*. identified a new carrier status or variant within the *ASGPR* gene dubbed ‘Gene X’ consisting of a 12-base pair deletion (del12). The study involved the sequencing of 2636 Icelanders in which identified variants were assigned into the genomes of 398,000 Icelanders, followed by testing the identified variants association with non-HDL cholesterol levels. Approximately 42,524 patients and 249,414 controls from five European populations were then assessed on the effects of a loss-of-function variant and the risk of CAD. A noncoding 12-base pair deletion was located in the *ASGR1* gene encoding the asialoglycoprotein. Upon translation, the variant is responsible for causing a frameshift mutation followed by a premature stop codon transforming the asialoglycoprotein receptor into a truncated protein, thus inhibiting receptor-glycoprotein complex establishment. Carriers of gene X have a 34% reduction in the risk of CAD including increased HDL and reduced LDL. The mechanism in which carrying a loss-of-function variant, however, and its association with exhibiting cardioprotective effects and cholesterol, is unknown [44].

## Inherited genetic susceptibility

Genotyping methods can be used to determine an individual's susceptibility to heritable conditions. For each of the conditions listed in this section, there are subgroups of patients showing phenotypes that cannot be explained only the basis of environmental factors and cardiovascular risk factors (Table 2).

**Table 2.** **List of genetic markers determining susceptibility to heritable conditions.**

**Condition**	**Gene name**	**Genetic variant (SNPs)**	**Allele**	**Association**	**Ref.**
Type III hyperlipoproteinemia	*Apolipoprotein E* (*APOE*)	rs429358 rs7412	T	Increased chance of inheriting Type III Hyperlipoproteinemia	Mahley *et al*. (2009) [45] Mahley *et al*. (1999) [46] Dong *et al*. (1996) [47] Brinton (2016) [48]

Atrial fibrillation	*Pituitary homeobox 2* (*PITX2*)	rs2200733	T	Increased chance of developing Atrial fibrillation	Wolf *et al*. (1991) [49] Nattel (2002) [50] Tao *et al*. (2014) [51] Gudbjartsson *et al*. (2007) [52] Syeda *et al*. (2017) [53] Wang *et al*. (2010) [54]

Coronary artery disease	*Cadherin 13 (CDH13*)	rs8055236	G	Increased risk of developing CAD	Won et al. (2015) [2] Wellcome Trust Case Control Consortium (WTCCC). (2007) [4] CARDIoGRAMplusC4D Consortium *et al*. (2013) [6] Erdmann *et al*. (2009) [56] Angelaopoulou *et al*. (2012) [57] Bressler *et al*. (2010) [58] Galkina and Ley (2007) [59] Khera and Kathiresan S (2017) [60]

	*Muscle RAS Oncogene Homolog (MRAS)*	rs9818870	T	–	

	*Methylenetetrahydrofolate Dehydrogenase (NADP+ Dependent) 1 Like (MTHFD1L)*	rs6922269	A	–	

	*SMAD, Mothers Against DPP Homolog 3 (SMAD3)*	rs17228212	C	–	

	*C-X-C Motif Chemokine Ligand 12 (CXCL12)*	rs501120	A	–	

	*Melanoma Inhibitory Activity Family Member 3 (MIA3)*	rs3008621	A	–	

	*Olfactory Receptors, Family 13 (OR13GI)*	rs2943634	C	–	

	*Adenomatous Polyposis Coli (APC)*	rs383830	T	–	

	*Lipoprotein Lipase (LPL)*	rs17411031	G	Decreased risk of developing CAD	

	*Cyclin Dependant Kinase Inhibitor 2B (CDKN2B)*	rs1333049	C	Increased risk of developing CAD	

Myocardial infarction	*C-X-C Motif Chemokine Ligand 12 (CXCL12)*	rs1746048	C	Increased risk of developing MI	Kathiresan *et al*. (2009) [5] Shiffman *et al*. (2005) [66] Samani *et al*. (2007) [67] Reilly *et al*. (2011) [68] Li *et al*. (2013) [69] Berger *et al*. (2015) [70] Farouk *et al*. (2010) [71] Huang *et al*. (2013) [72] Burke *et al*. (2017) [73] Almontashiri *et al*. (2014) [74]

	*Melanoma Inhibitory Activity Family Member 3 (MIA3)*	rs17465637	C	–	

	*Proprotein Convertase Subtilisin/Kexin Type 9 (PCSK9)*	rs11206510	T	–	

	*Phosphatase And Actin Regulator 1 (PHACTR1)*	rs12526453	C	–	

	*Sortilin 1 (SORT1)*	rs646776	A	–	

	Intergenic	rs9982601	T	–	

	Intergenic	rs10757278	G	–	

Peripheral arterial disease	*Cholinergic Receptor Nicotinic Alpha 3 (CHRNA3)*	rs1051730	T	Increased risk of developing peripheral arterial disease	Thorgeirsson *et al*. (2008) [76] Zintzaras and Zdoukopoulos (2009) [77] Han *et al*. (2015) [78]

Venous thrombosis	Methylenetetrahydrofolate reductase (*MTHFR*)	rs1801133	T	Increased risk of developing Venous thrombosis	Gao *et al*. (2012) [40] Cattaneo *et al*. (1997) [80] Margaglione *et al*. (1998) [81] Kim and Becker (2003) [82] Heijer and Lewington (2005) [83] Segal *et al*. (2009) [84] den Heijer *et al*. (1996) [85] Soria *et al*. (2000) [86] Soria *et al*. (2014) [87] Van Hylckama *et al*. (2014) [89]

	*Prothrombin*	rs1799963	A	–	

	*Factor V*	rs6025	A	–	

Hypertension	*Branched Chain Amino Acid Transaminase 1 (BCAT1)*	rs7961152	A	Increased risk of developing Hypertension	Kupper *et al*. (2006) [62] Hong *et al*. (2009) [63] Rhee *et al*. (2011) [64] Armando (2015) [65]

Sickle cell anemia	*Hemoglobin subunit beta (HBB)*	rs334	T	Homozygous carrier of the T allele is associated with Sickle Cell Anemia. Heterozygous carrier is associated with the sickle cell trait	Pauling *et al*. (1949) [3] Aidoo *et al*. (2002) [90]

### Type III HLP

Type III HLP is a rare genetic disorder which predisposes patients to early onset of atherosclerosis due to changes in lipid metabolism. This occurs when the protein apolipoprotein E is defective or deficient, resulting in less effective clearance of lipoproteins from the bloodstream (including chylomicron remnants, very LDL, intermediate density lipoproteins and a subclass of HDL) [45]. The disorder is therefore characterized by elevated plasma cholesterol and triglycerides [46], which may ultimately lead to accelerated CAD.

The inheritance of two recessive apoE2 alleles is the primary cause of Type III HLP. A single amino acid difference results in this isoform not binding efficiently to the LDL receptor in comparison to the E3 and E4 isoforms (0.01% of apoE3 or apoE4 activity) [47]. Although Type III HLP is characterized by the recessive apoE2 genotype, environmental factors that saturate or impair normal clearance of lipoproteins also contribute to the development of Type III HLP. Such factors include obesity, diabetes, hypothyroidism and oestrogen deficiency [45].

To reduce the chance of an individual developing Type III HLP, the intake of cholesterol and saturated fat in the diet should be reduced, and regular exercise should be undertaken. This would also apply once Type III HLP has been diagnosed to prevent the development of CAD. Additionally, a physician may also prescribe drugs to reduce blood lipid levels to prevent the onset of CAD, such as statins or fibrates [48].

### Familial AF

AF occurs due to an irregularity in the heart's electrical activity, causing an irregular and often rapid heartbeat. It causes irregular blood flow through the chambers of the heart which increases heart rate and blood pressure, and AF is a significant risk factor for stroke, particularly in the elderly [49]. Symptoms indicative of AF include palpitations, tiredness, shortness of breath and faintness.

The precise cause of AF is unclear, but several environmental and genetic factors are known to increase the risk of AF, and it can arise in individuals who are seemingly healthy (known as lone AF) [50]. Several cardiac conditions contribute to AF, including CAD, pericarditis, mitral valve disease, congenital heart disease, congestive heart failure, thyrotoxic heart disease and hypertension [50]. One of the known genetic risk factors for AF is a polymorphism of the gene *PITX2*, which produces a transcription factor known to regulate many genes including intercalated disc genes [51]. *PITX2* dysfunction results in electrical and structural remodeling and impaired repair of heart tissue, associated with a 1.4- to 1.5-times increased risk of developing AF [52–54].

Methods for preventing the onset of AF are similar to those usually associated with preventing heart disease including dietary changes and regular exercise. If an individual has developed AF they have an increased risk of stroke, so the prescription of anticoagulants may be necessary.

### Coronary artery disease

CAD is the leading cause of death worldwide, causing on average 190 deaths in the UK every day, mostly due to heart attack [55]. It occurs when cholesterol, fats, calcium and other substances build up in the coronary arteries, causing them to narrow. CAD can cause pain or discomfort (angina), breathlessness, nausea, restlessness, dizziness and tiredness.

An individual's risk of developing CAD has been shown to have a strong genetic component. An analysis that quantified heritability using updated genome-wide approaches estimated the heritability of CAD at 40–50% [2]. Polymorphisms in the following genes have been shown to influence the risk of CAD in large meta-analyses and genome-wide association studies: *CDH13*, *MRAS*, *MTHFD1L*, *SMAD3*, *CXCL12*, *MIA3*, *OR13G1*, *APC*, *CDKN2B*, *LPL* [4,6,56,57]. *CDH13* in particular, codes for a member of the cadherin superfamily, which protects vascular endothelial cells from apoptosis and is associated with resistance to atherosclerosis. Polymorphism of the *CDH13* gene can lead to protein dysfunction, which results in increased risk of CAD, possibly due to artery linings being more susceptible to damage by oxidative stress [58]. *MRAS* is a protein coding gene for a member of the Ras family, a signal transducer in multiple biochemical pathways, including cell growth and differentiation. Defects in this gene could disrupt adhesion signaling, which is important in the atherosclerotic process [59], so mutations in *MRAS* can influence the risk of developing CAD [56].

If genetic risk factors are present, it is imperative that an individual maintains a healthy lifestyle as a preventative measure to prevent CAD. Higher risk individuals may benefit from additional medications to reduce LDL cholesterol (e.g., statins), lower blood pressure or help prevent the formation of a blood clot (e.g., aspirin) [60].

### Hypertension

Hypertension is when blood pressure is higher than usual, and if untreated can increase the risk of life-threatening problems such as heart attacks and strokes. The normal range of blood pressure is considered to be between 90/60 and 120/80 mmHg, and hypertension is characterized as being over 140/90 mmHg [61].

The heritability of hypertension is cited to range from 30% to 60% [62]. One of these genetic components is a polymorphism of the *BCAT1* gene, which has been associated with increasing the risk of an individual developing hypertension in genome-wide studies [4,63,64]. The mechanism for this polymorphism is unknown, but it has been linked to increased salt sensitivity and is a marker of oxidative stress [65].

In order to reduce the risk of hypertension developing, it is important that a healthy lifestyle is maintained. A physician may also prescribe medication for hypertension, and these may include: ACE inhibitors, calcium channel blockers or β-blockers.

### Myocardial infarction

Most deaths from CAD are caused by a myocardial infarction (MI), commonly known as a heart attack. In the UK, there are nearly 200,000 hospital visits each year due to MI [55]. It occurs when heart muscle becomes irreversibly damaged, usually due to a thrombus in the coronary artery which restricts blood flow to the muscle, eventually leading to necrosis of the tissue. Symptoms of MI include: chest pain, shortness of breath, weakness and dizziness.

Many environmental factors can influence an individual's chance of developing MI, but it can also occur spontaneously when no other health issues are present. It has also been shown that MI is heritable, so there is a genetic basis for the risk of MI. Polymorphisms in the following genes or regions on the chromosome have been shown to influence the risk of MI in large meta-analyses and genome-wide association studies: *CXCL12*, *MIA3*, *PCSK9*, *PHACTR1*, *SORT1* and intergenic regions [5,66–70].

One particular pathway involved in susceptibility to MI is the *CXCL12* gene. This gene codes for a protein belonging to the CXC chemokine family and is a ligand for the CXCR4 receptor. This interaction has been linked to controlling various processes, such as embryonic development, hematopoiesis and angiogenesis; with animal models suggesting *CXCL12* has a function in vascular repair [71]. Polymorphisms of the *CXCL12* gene have been shown to increase the risk of early-onset MI [5] and CAD [72].

The *PCSK9* gene produces a protein that is secreted into plasma and binds to LDL receptors on hepatocytes to initiate endocytosis and degradation of the receptor by lysosomes. This reduces the number of LDL receptors on the hepatocyte surface, reducing the uptake of LDL particles from the bloodstream into the liver. Polymorphisms in this gene can cause loss of function, resulting in more LDL particles being removed from the blood and thus reducing the risk of MI [73]. Interestingly, *PCSK9* polymorphisms have a much greater effect on the risk of MI than the effect on LDL levels, suggesting *PCSK9* polymorphisms could be protective by a different mechanism [74].

Individuals with a high genetic risk can greatly reduce their risk of MI if adherence to a healthy lifestyle is maintained [9].

### Peripheral arterial disease

Peripheral artery disease (PAD) is a common but serious disease, which occurs when arteries other than those that supply blood to the heart become narrowed by atherosclerosis. Commonly, the arteries affected are those of the lower leg and most cases do not present any symptoms, but some individuals may have the following symptoms (usually in the legs): pain (intermittent claudication), weakness, change of skin color (blueness/paleness), and hair loss on lower body. Around one in five over 60s in the UK have PAD [75], and serious consequences may arise if left untreated, such as increased risk of MI or stroke, internal bleeding or critical limb ischemia.

A variant of the *CHRNA3* gene has been shown to increase the risk of developing PAD [76,77]. The exact mechanistic change is yet to be determined, however this SNP has been significantly associated with an individual's likelihood to smoke and therefore an increased likelihood of PAD and lung cancer [76,78]. Consequently, an individual with this SNP should be recommended to stop smoking as it may be that simply having the SNP is not the cause of an increased risk of PAD, but rather a result of the increased tendency to smoke.

### Venous thrombosis

VT occurs when blood clots begin to form in veins, usually veins in the legs (deep vein thrombosis). It is triggered by the activation of coagulation and thrombin-mediated fibrin deposition, which traps red blood cells and incorporates them into the clot [79]. This clot can break off or become dislodged and due to the pattern of the circulation, the clot may become lodged in the pulmonary arteries and cause pulmonary embolism, a life-threatening condition.

The cause of the initial coagulation trigger in VT is largely unknown, but an individual's genetics can confer an increased risk of developing VT. Polymorphisms in the following genes have been linked to an increased risk of VT: *Prothrombin*, *Factor V* and *MTHFR*, with some studies showing they have an additive effect [40,80–84]. These genes broadly control blood vessel damage and coagulation of blood. The *MTHFR* gene codes for an enzyme which processes folate. When function is altered due to a polymorphism, homocysteine levels can increase which damages blood vessels and therefore increasing the risk of VT [85]. Changes to usual blood clotting mechanisms can also increase risk of VT: prothrombin is usually cleaved to thrombin in the clotting process, polymorphisms in the prothrombin gene, however, can prevent this cleavage, increasing the concentration of prothrombin in the blood, resulting in an increased chance of developing a blood clot [86]. *Factor V* is a cofactor in the production of thrombin from prothrombin. Polymorphisms in this gene can cause hypercoagulability, as Factor V Leiden (the resultant mutated protein) cannot be inactivated by protein C, so continues to convert prothrombin to thrombin [87].

Other risk factors for VT include lifestyle factors, such as increasing age and being overweight or obese. The risk of VT also increases when an individual is inactive for long periods, such as after an operation, and certain conditions or treatments cause blood to clot more easily, increasing VT risk [88].

It has been shown that prediction of VT in high-risk individuals can be accurate when considering clinical and genetic predictors of the disease [88]. To decrease the risk of VT occurring, an individual should adopt healthy lifestyle changes as individuals with an increased genetic risk of VT have also been shown to have an increased risk of recurrence [89]. The treatments available for VT are mainly anticoagulants, including heparin, warfarin, rivaroxaban or apixaban.

### Sickle cell anemia

Sickle cell anemia (SCA) is a serious inherited condition which affects the shape and therefore function of red blood cells. This disease arises due to a homozygous genotype of *HbS*, a form of the hemoglobin gene, which causes hemoglobin proteins to aggregate so they cannot carry oxygen as efficiently. It also causes the shape of red blood cells to change (become sickled and rigid) so they can get lodged in small blood vessels leading to ischemia and infarction. Individuals with SCA can develop: anemia, fatigue, periodic episodes of pain (crises), swelling of hands and feet, frequent infections, delayed growth and visual problems.

SCA is caused by two copies of the faulty hemoglobin gene (*HbS*) [3]. Sickle cell trait (SCT) on the other hand, is when only one copy is present, making the individual a carrier of the faulty allele. SCT is not associated with the severe symptoms which occur in SCA, and is protective against malaria, which is thought to be the reason the HbS allele is common in malaria endemic regions [90].

As SCA is a purely genetic condition, it is a lifelong diagnosis, so at present the only treatments available work to manage the manifestations of the disease. Examples include hydroxycarbamide to reduce crises, supplements of iron to treat anemia and prophylactic antibiotics. If an individual has sickle cell trait, they are at risk of passing this onto their offspring if their partner also has sickle cell trait. The risk of passing on SCA and SCT is 25 and 50%, respectively, and there is a 25% chance the child will have neither SCT or SCA. Although such individuals may not necessarily present any symptoms, early genetic testing is important for identifying copies of faulty genes and to minimize the risk of passing the disease onto offspring.

## Pharmacogenetics & future potential

Pharmacogenetics is often defined as the study of drug metabolic pathways in relation to specific genes, that ultimately affect an individual's response to certain drugs [91,92]. Many of the conditions mentioned above have treatment regimes in place. While genotyping is key in identifying susceptibility to inherited conditions, identifying an individual's metabolic pathway can make way for tailored, bespoke treatments, specific to ones’ unique genetic profile. Ultimately reducing costs, ‘trial and error’ of drug prescriptions and adverse effects, whilst improving patient compliance within a clinical setting.

## Conclusion

CVDs are the leading cause of death worldwide and the development of such diseases involves a complex interaction between the genome and the environment. Understanding an individual's genetic profile along with lifestyle factors can determine their ability to metabolize certain nutrients and the direct influence this has on heart health. This knowledge is key in preventing such diseases and for promoting a better quality of life.

Identifying polymorphisms within individuals has enabled early identification of inherited conditions. This has relevance to the present day in which we are progressing toward an era of personalized healthcare and the increased availability of -*omics* technologies. Genotyping methods can be used to determine an individual's susceptibility to heritable conditions and how certain genetic risk factors can be countered by incorporating prevention plans and adopting a healthier lifestyle, thus enabling better preparation for the future. Early identification is key in preventing the onset of heart disorders and genotyping can allow for the introduction of appropriate interventions that contribute to decreased risk of heart disease.

In summary, this review expands our understanding of CVDs and how such conditions arise. Environmental and lifestyle factors play a huge part in the increased risk of developing CVD. In addition, one's unique genetic makeup can also be responsible for the increased susceptibility and pathogenesis of CVDs, while time and effort continues to be invested into fully understanding the genes and DNA variants that contribute to this.

## Future perspective

We are already seeing a surge in healthy individuals moving to personalize their healthcare and decode their genes, either out of interest or to get answers for questions they might have in relation to an imminent family history with a certain disease. Additionally, individuals are seeking for a tailored/bespoke treatment regimen that suits their needs and fits their genetic profile.

Over the next few years we will certainly see a surge in people being referred to personalize their healthcare, as it proves to be the only route to save money and enhance quality of care. However, this will not happen before a spike in genomics education becomes more prevalent and doctors become more engaged and aware of the basis of such tests, and how to use the retrieved data in conjunction with their patients’ self-reported information. New models to engage medical practitioners in such transformational shift would need to be put in place to educate and incentive medical practitioners to take part in this movement.

**Executive summary**

**Background**

- Cardiovascular diseases (CVDs), characterized by diseases of the heart and blood vessels, are the number one cause of death globally representing 31% of all global deaths in 2015.
- The identification of polymorphisms that confer an increased risk for CVDs, and that determine responses to CVD drugs, will become increasingly important as new -omics technologies take us forward into the personalized medicine era.

**Diet & nutrition**

- Genetic markers can predispose an individual to metabolize certain nutrients differently to others and thus have an impact on CVD risk.

**Inherited conditions**

- Genotyping methods can be used to determine an individual's susceptibility to heritable conditions.
- Early genetic testing is key in preventing the onset of heart disorders and implementing the correct interventions that can reduce the chances of developing such conditions.

**Pharmacogenetics & future potential**

- Identifying an individual's metabolic pathway can make way for tailored/bespoke treatments, specific to ones’ unique genetic profile.

**Conclusion**

- Environmental and lifestyle factors play a huge part in the increased risk of developing CVD.
- One's unique genetic makeup can also be responsible for the increased susceptibility and pathogenesis of CVDs.
